# Pathophysiology of cellulite: Possible involvement of selective endotoxemia

**DOI:** 10.1111/obr.13517

**Published:** 2022-10-26

**Authors:** Ilja L. Kruglikov, Philipp E. Scherer

**Affiliations:** ^1^ Scientific Department Wellcomet GmbH Karlsruhe Germany; ^2^ Touchstone Diabetes Center, Department of Internal Medicine University of Texas Southwestern Medical Center Dallas Texas 75390‐8549 USA

**Keywords:** lipedema, lipopolysaccharide, MMP14, MUSE cells

## Abstract

The most relevant hallmarks of cellulite include a massive protrusion of superficial adipose tissue into the dermis, reduced expression of the extracellular glycoprotein fibulin‐3, and an unusually high presence of MUSE cells in gluteofemoral white adipose tissue (gfWAT) that displays cellulite. Also typical for this condition is the hypertrophic nature of the underlying adipose tissue, the interaction of adipocytes with sweat glands, and dysfunctional lymph and blood circulation as well as a low‐grade inflammation in the areas of gfWAT affected by cellulite. Here, we propose a new pathophysiology of cellulite, which connects this skin condition with selective accumulation of endogenous lipopolysaccharides (LPS) in gfWAT. The accumulation of LPS within a specific WAT depot has so far not been considered as a possible pathophysiological mechanism triggering localized WAT modifications, but may very well be involved in conditions such as cellulite and, secondary to that, lipedema.

## 1 INTRODUCTION

Cellulite is a dimpled lumpy skin appearance in the gluteofemoral body area occurring almost exclusively in females. This chronic skin condition is widespread and affects more than 85% of females over the age of 20. Cellulite does not necessarily correlate with BMI; its onset can be seen as early as during puberty. It does not demonstrate a significant association with lipedema, which is another GF‐related disorder, but much more severe in its manifestations, with reduced incidence rate and unknown pathophysiology, apparent mostly as a dramatic expansion of the subcutaneous white adipose tissue (sWAT).^1^, ^2^ However, because both cellulite and lipedema appear mainly in the gluteofemoral areas of women, we cannot exclude that these two conditions reflect consecutive stages of one and the same process. This makes the understanding of the pathophysiology of cellulite potentially important for lipedema.

Various pathophysiologies of cellulite were proposed in the past. These include connecting this skin condition to lobular structures or to the sexually dimorphic architecture of the fibrous septa of sWAT. Other explanations include modifications in lymphatic and blood circulation, or it has been related to the phenomenon of low‐level chronic inflammation with deposition of glycosaminoglycans in affected WAT.^3^ However, none of these proposed mechanisms can explain the most pronounced morphological alterations observed in the cellulite skin, namely, the distinct protrusions of the underlying adipose tissue into the dermis.

Under physiological conditions, adipocytes in gluteofemoral WAT (gfWAT) are significantly larger than their abdominal counterparts.^4^, ^5^ Moreover, gfWAT in cellulite is of a hypertrophic nature (i.e., adipocyte cell size is increased) and is significantly expanded compared with a non‐cellulite skin.^6^, ^7^ Whereas lumpy structures of the dermal–hypodermal junctions are a physiological feature in gfWAT,^8^ the strongly increased protrusions of adipose tissue into the dermis through dermal–hypodermal junctions are a histological hallmark of cellulite.^9^ Earlier magnetic resonance imaging (MRI)‐based analysis revealed that the index of irregularity of dermal–hypodermal junctions, describing the relative height of adipose protrusions into the dermis, is bigger in females with cellulite than in females without cellulite or in males.^6^, ^10^ More recent histological analysis with hematoxylin stains confirmed pronounced differences in the amounts of adipose tissue protrusions into the dermis (the relative areas covered by perilipin‐positive adipose tissue), which were 10.5% ± 1.1% in females with cellulite, 5.0% ± 0.9% in females without cellulite, and 3.9% ± 1.1% in males.^9^ Reduction of the contact surface area between dermis and WAT with a corresponding reduction of herniations correlated with optical improvement of the cellulite skin.^11^

Pronounced protrusions of adipose tissue into the dermis can be caused either by reduced levels of adhesion between skin and WAT on the dermal–hypodermal junctions or by special properties of the superficial adipose tissue in cellulite skin, allowing its higher invasiveness. Here, we analyze recent results concerning the superficial adipose tissue in humans as well as the new morphological findings in cellulite skin and underlying adipose tissue. We propose a new pathophysiology of this skin condition, which can explain the appearance of WAT protrusions into the dermis.

## 2 SKIN‐ASSOCIATED ADIPOSE TISSUE: A SPECIAL FAT LAYER CONTRIBUTING TO CELLULITE

Murine adipose tissue consists of two different layers—dermal white adipose tissue (dWAT) and subcutaneous adipose tissue (sWAT)—separated by the *panniculus carnosus*, a layer of striated muscle cells.^12^ Whereas there is no anatomical demarcation for dWAT in human skin analogous to the *panniculus carnosus* in rodents, there are some structures equivalent to this layer of striated muscle cells in humans, referred to as *superficial musculo‐aponeurotic system* (SMAS) in the face, *platysma* in the neck, and *fascia superficialis* or *Camper's fascia* in the abdomen and the thighs.^13^ To avoid possible confusion, some authors proposed to name dWAT in humans as skin‐associated adipose tissue (SAAT)^14^ or skin‐associated fat (SAF).^15^ This depot corresponds in the thighs to the superficial WAT located above the superficial fascia and is distinct in its anatomical structure and metabolic properties from the underlying deeper WAT layer, located between superficial fascia and deep fascia.^16^ SAAT has a clear lobular structure and pronounced fibrous septa composed of elastic fibers (i.e., extracellular matrix macromolecules comprising an elastin core surrounded by a mantle of fibrillin‐rich microfibrils) and collagen fibers, producing the typical polygonal‐oval lobules of mature adipocytes and connecting dermis with superficial fascia. The lobular structure of SAAT is not a unique property of the gfWAT but was also observed in other areas.^13^, ^17^ Contrary to this, underlying deep sWAT is characterized by smaller, less well‐defined, and flat lobules as well as by a loose fibrous septa connected to the deep fascia.^18^

Chemical shift MRI, allowing to separate water and fat, was applied to investigate the dWAT in rodents and SAAT depots in humans.^15^, ^19^ The thickness of SAAT demonstrates a high interindividual variability and was reported to be about 10.05 ± 2.45 mm in females versus 6.27 ± 1.89 mm in males, thus demonstrating a clear sexual dimorphism. Importantly, the thickness of this layer is independent of BMI in males and only marginally affected by this parameter in females.^15^ Earlier, SAAT and sWAT thicknesses were found to be 8.41 ± 1.61/24.81 ± 4.90 mm and 4.03 ± 1.26/4.31 ± 1.82 mm in females with and without cellulite, respectively.^6^ Additionally, it was shown that females have much weaker fibrous septa than males, which thus can be deformed and damaged by application of even relatively low mechanical forces.^20^ According to these results, the SAAT layer doubles its thickness in cellulite, whereas sWAT expands more than five times. Such significant expansion of both SAAT and sWAT in cellulite skin can significantly modify the mechanical behavior of the composite skin/WAT under loading, leading to its bending deformations.^21^

The composite skin/WAT consists of different layers having various thicknesses and mechanical properties. Some of these layers are stiffer (e.g., the *stratum corneum*), whereas others (especially, SAAT and sWAT) are very compliant. Mechanical behavior and structural stability of such layered composites are strongly dependent on such characteristics as thickness ratio between the neighboring layers and their bending and tensile stiffness as well as adhesive strength between the layers.^21^ Appearance of two thick but mechanically weak WAT layers (SAAT and sWAT) with good adhesion to the superficial and deep fascia significantly modifies the mechanical stability of such layered structures; this effect will be especially pronounced in the case of mechanical loading directed perpendicular to the skin surface.^21^ To counteract this effect, the mechanical stiffness of expanded SAAT and sWAT has to be increased, which can be achieved through reinforcement of these WATs with collagen networks.

## 3 REDUCTION OF FIBULIN‐3 IN CELLULITE SKIN AND THE ROLE OF MMP14

The extracellular glycoprotein fibulin‐3 (EFEMP1) was found to be significantly reduced, both in cellulite skin and in fibrous septa.^9^ Fibulin‐3 deficiency is connected to a deficit of elastic fibers localized to fascia connective tissue, which normally leads to the development of weakness of the fascia, tissue herniation and even to prolapse.^22^

Fibulin‐3 is a substrate for matrix metalloproteinase 14 (MMP14).^23^ A substantial reduction of fibulin‐3 in cellulite skin can be caused by its enhanced shedding through MMP14. MMP14 is the main pericellular collagenase in adipose tissue and the only MMP that directly promotes cellular invasion in collagen‐rich matrices.^24^ Based on these properties, this MMP may be substantially involved in the generation of the protrusions of superficial adipose tissue into the collagen‐rich dermis.

We have demonstrated that MMP14 is typically overexpressed in hypertrophic WAT.^25^ MMP14 cleaves the α3 chain of type VI collagen, thereby releasing endotrophin that is consequently more abundant in hypertrophic WAT where it triggers fibrosis and inflammation.^26^ Such increased activity of MMP14 should be of primary importance in cellulite characterized by hypertrophic gluteofemoral adipose tissue.^6^, ^7^ Accordingly, local overexpression of MMP14 in hypertrophic WAT would reduce elastic networks in the skin and in the underlying WAT. This will lead to a reduction of the papillary dermis and *rete ridges*, and simultaneously cleave fibulin‐3, thus providing conditions for protrusion of the superficial adipose tissue into the dermis. Indeed, cellulite skin demonstrates a significant reduction of the *rete ridges* and a corresponding thinning of the papillary dermis.^9^

Enhanced expression of MMP14 also promotes expression of MMP2, cleaving alarmin (S100A9), which leads to a reduction of the inflammatory response.^27^ Additionally, MMP14 sheds the hyaluronan (HA) receptor CD44, thus modifying HA content and cell adhesion as well as promoting cellular invasion.^24^, ^28^ MMP14 co‐localizes with caveolin‐1 (CAV1) in plasma membranes of different cells.^2^ On the other hand, MMP14 is a target for the transcriptional factor prospero homeobox 1 (PROX1), which is a master regulator for the lymphatic system.^2^ Because CAV1, HA and PROX1 are all affected through their interaction with MMP14, mechanical properties of the layered composite skin/sWAT and content of glycosaminoglycans as well as the lymphatic circulation in this area are modified. Such modifications have indeed been seen in the context of cellulite and have been reported by different authors.

## 4 SOME SPECIAL FEATURES OF GFWAT IN THE CONTEXT OF CELLULITE

Even under normal physiological conditions, gfWAT is somewhat unique as judged by its enhanced expression of stearoyl‐CoA desaturase (SCD1), which is involved in the conversion of palmitic acid into palmitoleic acid. This activity is significantly higher in gfWAT than in any other WAT depots.^29^ SCD1 has two main substrates—stearic and palmitic acids, the conversion of which generates oleic and palmitoleic acid, respectively. Overexpression of SCD1 leads to an increased ratio of monounsaturated to saturated fatty acids in cell membranes, resulting in enhanced membrane fluidity and cellular invasiveness.^30^ Accordingly, the disproportionate expression of SCD1 observed in gfWAT will lead to an increased production of monounsaturated palmitoleic acid in this area of the body. Indeed, gfWAT is the main source for palmitoleic acid, even under normal physiological conditions.^29^, ^31^ This effect may be connected to the known anti‐inflammatory impact of palmitoleic acid, ameliorating tissue inflammation induced by palmitic acid.^32^

Proteomic analysis reveals an unusually high presence of “multi‐lineage‐differentiating stress‐enduring (MUSE) cells” in gfWAT prone to cellulite.^3^ MUSE cells are noncancerous, stress‐tolerant pluripotent human mesenchymal stromal cells (hMSC). These cells can differentiate into adipocytes by exposure to different factors. They also express estrogen receptors and are known to play an important role in tissue repair and regeneration. In gfWAT prone to cellulite, these cells constitute about 85%–90% of the whole mesenchymal stromal cell population, demonstrating a drastic difference to the relatively low percentage of these cells usually found under normal physiological conditions. Moreover, other reports correlate the number of MUSE cells directly with the clinical grade of cellulite.^3^

MUSE cells express specific nuclear receptors, such as liver X receptor (LXR) and farnesoid X receptor (FXR), which dimerize with retinoid X receptor (RXR) producing LXR/RXR and FXR/RXR signaling complexes.^33^ Whereas LXRα directly promotes lipogenesis through induction of fatty acid synthase (FASN) and SCD1, FXR downregulates lipogenesis by suppressing FASN and SCD1.^34^ Moreover, LXR agonists trigger SCD1 expression in mesenchymal stromal cells, providing a drastic reduction of the inflammatory response and in palmitate‐induced cell death.^35^ These effects are achieved by a downregulation of pro‐inflammatory cytokines and an upregulation of the anti‐inflammatory cytokine IL‐10.^36^ Whereas this topic was not investigated in depth, it is safe to assume that gfWAT associated with cellulite reflects an imbalance between overexpression of LXRs and underexpression of FXRs, thereby leading to enhanced lipogenesis and overproduction of SCD1 in this depot.

Very recently, the high stress tolerance characteristic of MUSE cells was connected to a potently enhanced activation of the *CCNA2* gene, encoding the cyclin A2 protein.^37^ Cyclin A2 regulates the cell cycle through its interaction with two different cyclin‐dependent kinases (CDKs) in the S and G2 phases of the cell cycle, whereas the depletion of cyclin A2 induces cell cycle arrest.^38^ Transformation of hMSCs into MUSE cells in cellulite gfWAT means that these cells abandon cell cycle arrest and start to proliferate.

Remarkably, cellulite skin has an additional histological hallmark, which is distinct from other fat depots: a strong spatial association and paracrine interaction of mature adipocytes with sweat glands.^3^, ^39^ Such associations of mature adipocytes with sweat glands is a unique feature in adenolipoma—a rare benign tumor mainly observed in gfWAT.^40^ Adenolipoma has a typical lobular structure; its lobules are bigger than the corresponding structures in sWAT under normal physiological conditions. Sodium chloride is the main component of sweat gland secretion. It is well established that overloading of adipose tissue with salts leads to activation of the renin–angiotensin–aldosterone system and release of Ang II, which induces activation of inflammatory cytokines and enhances adipogenesis/lipogenesis.^41^ To induce this effect, there should be “leakage” of sweat from the gland. Such leakage is typical in atopic dermatitis and was associated with decreased expression of claudins, especially of claudin‐3—the major component of tight junctions, both in sweat ducts and secretory coils.^42^ Interestingly, loss of tight junction proteins is mediated by the downregulation of CAV1, whereas upregulation of CAV1 can strongly counteract this destructive process.^43^

**FIGURE 1:**
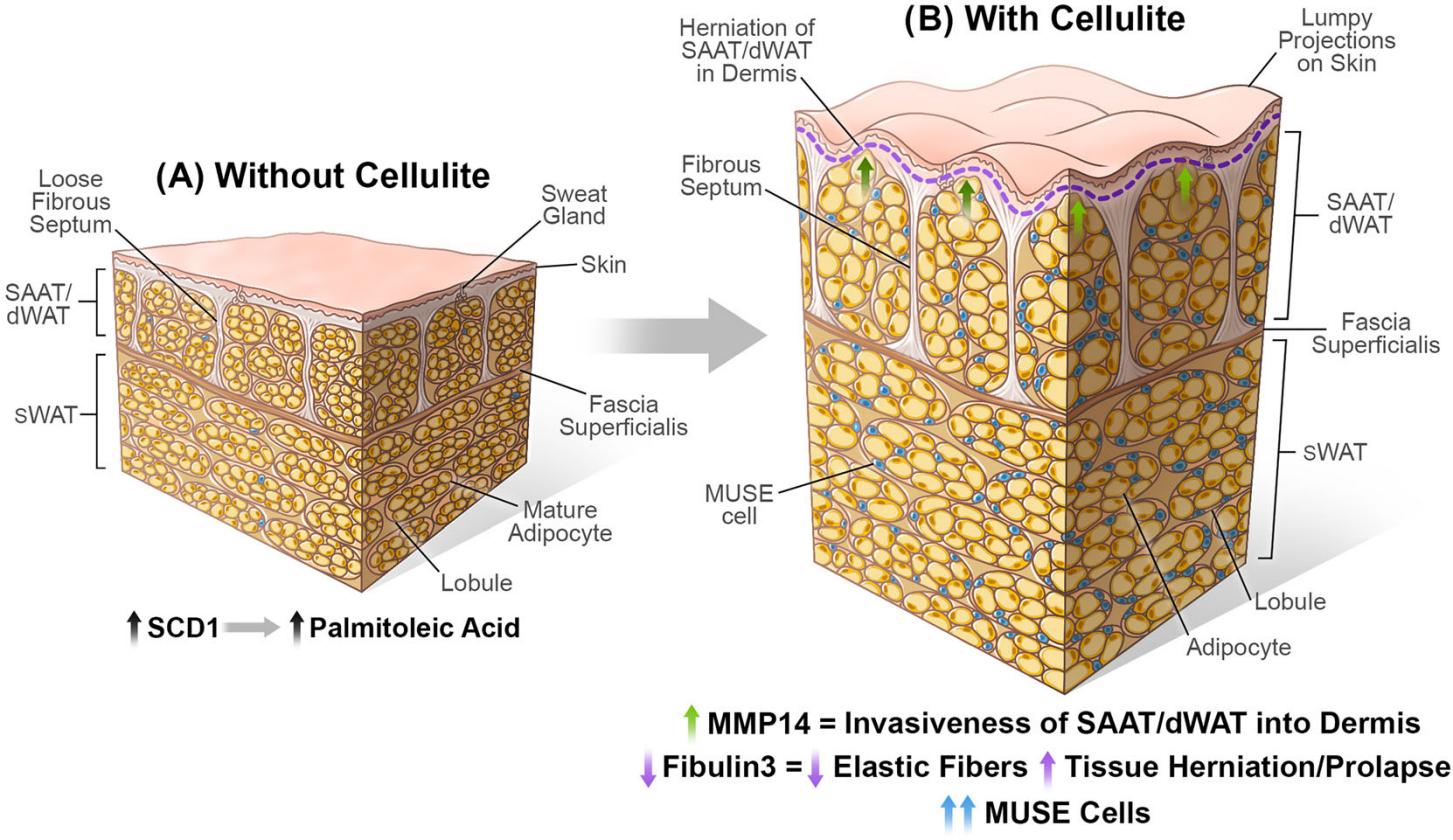
Gluteofemoral adipose tissue (gfWAT) with and without cellulite. (A) gfWAT without cellulite: SCD1 (stearoyl‐CoA desaturase) and resulting palmitoleic acids are generally increased in gluteofemoral adipose tissue (gfWAT). (B) gfWAT with cellulite: The thicknesses of SAAT and sWAT increase, respectively, about twofold and sixfold; MMP14 is strongly increased, producing conditions for the invasiveness of SAAT/dWAT into the dermis; fibulin‐3 is strongly diminished, causing a reduction of elastic fibers and conditions for tissue herniation/prolapse; the number of the MUSE cells is dramatically increased.

These special features of gfWAT are summarized in Figure 1.

## 5 SELECTIVE ENDOTOXEMIA IN GFWAT DEPOT AS A POTENTIAL CONTRIBUTOR TO CELLULITE

Both the overexpression of SCD1 in gfWAT and the accumulation of MUSE cells in cellulite skin point to the existence of an endogenous source of inflammation, even in physiologically intact gfWAT. It can be assumed that the reason for pro‐inflammatory and pro‐cell‐cycle‐arrest conditions in gfWAT could be a selective, low‐level accumulation of bacteria‐derived lipopolysaccharides (LPS). Indeed, we know that LXR activation enhances Toll‐like receptor 4 (TLR4) protein expression (the main receptor for LPS) and also regulates the TLR4‐mediated response to LPS in human macrophages.^44^ Moreover, activation of both LXRs/RXR and FXRs/RXR pathways protects different tissues from injury induced by LPS,^45^, ^46^ whereas the pro‐inflammatory cytokines induced by LPS downregulate FXR expression.^47^ Importantly, hMSCs undergo senescence under prolonged LPS stimulation,^48^ and undifferentiated cells exposed to LPS undergo cell cycle arrest,^49^ which coincides with the appearance of MUSE cells. In line with this observation, a reduction of SCD1 activates TLR4 expression in adipose tissue, elevating the LPS‐induced inflammatory response in macrophages^50^; correspondingly, overexpression of SCD1 in gfWAT will counteract this activation. Enhanced expression of TLR4 receptors in gfWAT will make this fat depot more sensitive to low tissue concentrations of LPS, thereby increasing the risk of a local inflammation.

The differentiation of preadipocytes into adipocytes is associated with a high level of expression of the antimicrobial peptide cathelicidin (in humans, LL‐37) (14) that can potently neutralize LPS.^51^ Increased expression of cathelicidin is associated with an expansion of the dWAT in a murine model.^52^ It is likely that an analogous reaction exists in humans and that the expansion of SAAT and sWAT in gfWAT in cellulite can be linked to endogenous accumulation of LPS in this fat depot with an enhanced tissue sensitivity to LPS through high‐level TLR4 expression.

LPS induces the disruption of tight junctions in different tissues, thereby causing a breakdown in their barrier function. At least in some epithelial tissues, this effect is connected to reduced expression of claudins.^53^ Thus, the accumulation of endogenous LPS in gfWAT can lead to leakage of sweat from the sweat gland, leading to modification of surrounding adipose tissue causing its inflammation and expansion through adipogenesis/lipogenesis.

It is widely accepted that metabolic endotoxemia, that is, the translocation of LPS from intestine into circulation caused by decreased barrier function of the intestine, induces low‐grade inflammation in different tissues (including adipose tissue) through activation of TLR4, thereby playing a permissive role for WAT expansion and contributing to the development of obesity and associated diabetes.^54^, ^55^ Remarkably, endotoxemia induces the expression of pro‐inflammatory cytokine IL‐6 mainly from WAT, and its expression strongly increases with aging.^56^ Such enhanced expression of IL‐6 is directly connected to MMP14 overexpression in the affected tissue area through downregulation of p53, which also leads to the development of an invasive cellular phenotype.^57^

LPS can also enhance preadipocyte proliferation and adipogenesis.^58^ Mature adipocytes treated in vitro with LPS demonstrate 3.7‐fold higher accumulation of triacylglycerols than their non‐treated controls.^59^ Translated to an in vivo setting, this may cause a hypertrophic expansion of the affected gfWAT. At least in lean females and at moderate LPS concentrations, sWAT demonstrates a much more pronounced reaction to LPS than visceral WAT, mainly due to a stronger NF‐κB pathway activation.^60^ In fact, it was shown that NF‐κB directly regulates the expression levels of all three lipid desaturases (including SCD1), and vice versa, inhibition of desaturases blocks the NF‐κB pathway,^61^ which consequently leads to an attenuation of the pro‐inflammatory effect of LPS on WAT.

Additionally, LPS stimulates expression of FASN and significantly enhances palmitic acid synthesis in macrophages.^62^ Remarkably, combination of LPS with palmitic acid demonstrates a synergistic impact upon induction of the monocyte chemoattractant protein‐1, thus amplifying inflammation in WAT.^63^ Similar interactions between LPS and palmitic acid were also reported in microglial activation and neuroinflammatory responses.^64^

Moreover, LPS modulates the collagen synthesis in dermal fibroblasts in a dose‐dependent manner—low/high doses of LPS increase/decrease collagen production in these cells.^65^ Especially, LPS can modulate the production of COL6α3, which along with enhanced expression of MMP14 in hypertrophic WAT leads to an increased production of endotrophin, thus enhancing fibrosis in affected adipose tissue. This effect was observed in murine lungs after intraperitoneal injection of LPS^66^ as well as in WAT, where it was connected with induced expression of dermatopontin.^67^

Whereas it was shown that application of LPS in high doses suppresses the tissue expression of MMP14,^27^ dose‐dependent influence of LPS on the expression of MMP14 was not investigated in detail so far. On the other hand, low‐grade endotoxemia leads to hypertrophic expansion of WAT, which correlates with enhanced expression of MMP14. This means that LPS influences MMP14 in a dose‐dependent manner similar to that observed for collagen synthesis.

The biodistribution of LPS and its main receptor, TLR4, in various regional subcutaneous fat depots has not yet been investigated in detail. However, accumulation of LPS is not passively dependent on a given expression of the TLR4 receptors in the tissue because LPS can up to several times increase the TLR4 expression in a dose‐dependent manner, which was demonstrated in 3T3‐L1 cells in vitro as well as in murine hypertrophic scar models and which can lead to scarring through induction of collagen synthesis and TGF‐β pathway.^68^

**FIGURE 2:**
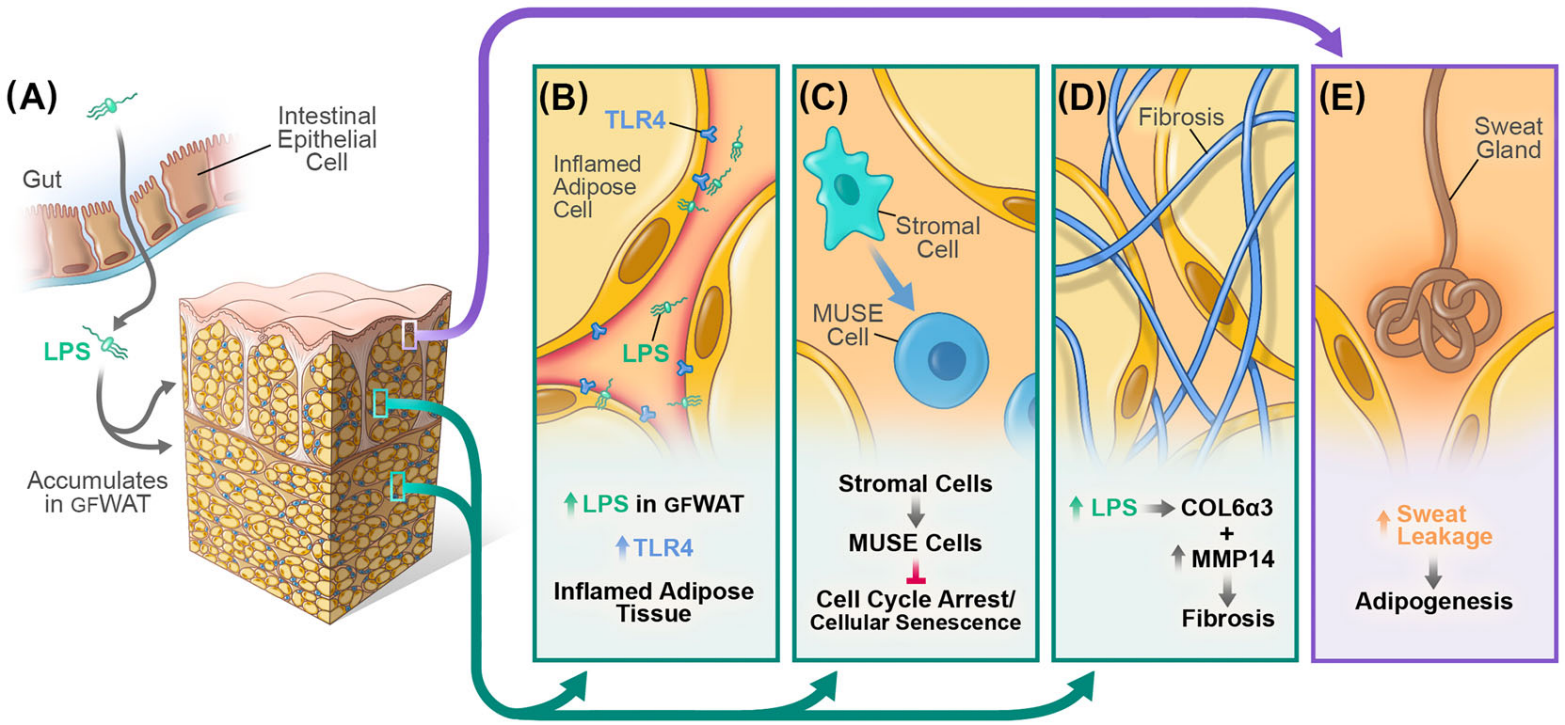
Leakage of LPS from the gut and its selective accumulation in gfWAT. Lipopolysaccharides leak from the gut and selectively accumulate in low doses in gfWAT. This accumulation is connected with preexisting and induced TLR4 receptors in gfWAT. This provides conditions for inflamed adipose cells and overall inflamed gfWAT. Under conditions of low‐grade inflammation, stromal cells transform into MUSE cells to counteract the cell cycle arrest and cellular senescence. LPS induces production of endotrophin (COL6α3) that together with increased MMP14 provides conditions for local fibrosis in gfWAT. Sweat glands in SAAT/dWAT have a strong spatial association and paracrine interaction with mature adipocytes; leaking sweat components induce adipogenesis in surrounding gfWAT.

Selective accumulation of LPS in gfWAT and its role in cellulite are summarized in Figure 2.

## 6 DISCUSSION

There are several hallmarks of cellulite, which we can better understand when we look at the pathophysiology of this skin condition in detail. The most relevant aspects of these hallmarks include a massive protrusion of superficial adipose tissue into dermis, reduced expression of the extracellular glycoprotein fibulin‐3, and an unusually high presence of MUSE cells in gfWAT that displays cellulite. Also typical for this condition is the hypertrophic nature of the underlying adipose tissue, the interaction of adipocytes with sweat glands (with production of structural units which are not seen in other WATs), and dysfunctional lymph and blood circulation as well as a low‐grade inflammation in the areas of gfWAT affected by cellulite.

Protrusions of gfWAT into the dermis seen in cellulite can be connected with the special properties of SAAT, which is a human adipocyte layer similar to the dWAT layer in rodents. This layer structurally and physiologically differs from its deeper counterpart (sWAT) and has the ability to quickly expand in response to a number of different stimuli, including infection. It also demonstrates a pronounced sexual dimorphism, being much thicker in females than in males.^12^ Adipocytes from such superficial adipose layers demonstrate potential for de‐ and redifferentiation, quickly changing their phenotype (14). SAAT develops protrusions into the dermis even under normal physiological conditions, producing “dermal cones” around the hair follicles, whose appearance correlates with the ability to undergo hypertrophic scarring.^12^ Hypertrophic SAAT and sWAT in cellulite lead to enhanced expression of MMP14—the main pericellular collagenase in adipose tissue directly promoting cellular invasion in the collagen‐rich matrix of the dermis. The highly invasive activity of superficial adipocytes in combination with the overexpression of MMP14 near the dermal–hypodermal junction provide the conditions for effective protrusions of SAAT into the dermis in cellulite skin. Because MMP14 cleaves fibulin‐3, such a process will cause destruction of the elastic network and prompt a significant reduction of the papillary dermis and *rete ridges*, which is indeed observed in cellulite skin.^9^

Intact gfWAT is characterized initially by high levels of SCD1 and palmitoleic acid production pointing to a possible defensive mechanism against inflammation. Cellulite gfWAT additionally demonstrates unusually high abundance of stress MUSE cells that express the liver X receptors (known to upregulate SCD1 expression) and displaying high expression of *CCNA2*, which is involved in cell cycle control. gfWAT in cellulite counteracts endogenous inflammatory stimuli and also provides factors, leading to cell cycle arrest. Taking into account the spatial location of gfWAT, such behavior is also connected with the accumulation of endogenous LPS in gfWAT. LPS accumulation induces a disruption of the barrier function in the sweat glands that are highly abundant in gfWAT, causing sweat leakage with a resulting impact of the leaked salt on the inflammation and lipogenesis in gfWAT.

Metabolic endotoxemia is a widely accepted pathophysiological factor in generalized adiposity and diabetes. However, the accumulation of LPS within a specific WAT depot has so far not been considered as a possible pathophysiological mechanism triggering localized WAT modifications, but may very well be involved in such conditions as lipedema or cellulite. Here, we propose that the gfWAT depot provides conditions for local activation of TLR4 receptors. This makes this tissue more sensitive to low doses of LPS present in gfWAT and will induce a low‐grade inflammation and promote irregular accumulation of collagen as well as development of fibrosis in this fat depot.

Whereas such low‐grade gfWAT‐specific endotoxemia should be a common effect leading to its low‐grade inflammation accompanied by fibrosis and enhanced protrusions into the dermis, further increase of endotoxemia level in gfWAT must lead to the uncontrollable expansion of this tissue observed by lipedema. This paradoxical situation can make cellulite a pre‐stage of lipedema—a topic that should be carefully addressed in future research.

## CONFLICT OF INTEREST

ILK is the managing partner of Wellcomet GmbH. Wellcomet GmbH provided support in the form of salaries for ILK but did not have any additional role in decision to publish or preparation of the manuscript. The commercial affiliation of ILK with Wellcomet GmbH does not alter the adherence to all journal policies on sharing data and materials. PES declares no conflict of interest.
